# Methanol-Tolerant Platinum-Palladium Catalyst Supported on Nitrogen-Doped Carbon Nanofiber for High Concentration Direct Methanol Fuel Cells

**DOI:** 10.3390/nano6080148

**Published:** 2016-08-15

**Authors:** Jiyoung Kim, Jin-Sung Jang, Dong-Hyun Peck, Byungrok Lee, Seong-Ho Yoon, Doo-Hwan Jung

**Affiliations:** 1 Fuel Cell Research Center, Korea Institute of Energy Research (KIER), Daejeon 305-343, Korea; jwjjs@hanmail.net (J.-S.J.); dhpeck@kier.re.kr (D.-H.P.); brlee@kier.re.kr (B.L.); 2 Advanced Energy Technology and System, University of Science and Technology (UST), Daejeon 305-333, Korea; 3 Institute for Materials Chemistry and Engineering, Kyushu University, Fukuoka 816-8580, Japan; yoon@cm.kyushu-u.ac.jp

**Keywords:** nitrogen doped carbon nanofiber, methanol tolerant, oxygen reduction reaction, direct methanol fuel cell

## Abstract

Pt-Pd catalyst supported on nitrogen-doped carbon nanofiber (N-CNF) was prepared and evaluated as a cathode electrode of the direct methanol fuel cell (DMFC). The N-CNF, which was directly synthesized by the catalytic chemical vapor deposition from acetonitrile at 640 °C, was verified as having a change of electrochemical surface properties such as oxygen reduction reaction (ORR) activities and the electrochemical double layer compared with common carbon black (CB). To attain the competitive oxygen reduction reaction activity with methanol tolerance, the Pt and Pd metals were supported on the CB or the N-CNF. The physical and electrochemical characteristics of the N-CNF–supported Pt-Pd catalyst were examined and compared with catalyst supported on the CB. In addition, DMFC single cells using these catalysts as the cathode electrode were applied to obtain I-V polarization curves and constant current operating performances with high-concentration methanol as the fuel. Pt-Pd catalysts had obvious ORR activity even in the presence of methanol. The higher power density was obtained at all the methanol concentrations when it applied to the membrane electrode assembly (MEA) of the DMFC. When the N-CNF is used as the catalyst support material, a better performance with high-concentration methanol is expected.

## 1. Introduction

A direct methanol fuel cell (DMFC) is closely related to a polymer electrolyte fuel cell (PEFC). While the PEFC uses hydrogen gas with enough moisture as the fuel, the DMFC is supplied with a diluted methanol solution. Methanol is directly decomposed into CO_2_, protons and electrons at the anode side. Protons passed through the polymer membrane and electrons through the external circuit combine with oxygen in air and produce water at the cathode side. During the occurrence of the methanol oxidation at the anode electrode and the oxygen reduction at the cathode electrode, the electrons are passed through the external circuit and generate electricity. The DMFC has significant advantages such as high energy density, a system with simple configuration and convenient transportation of fuel due to the use of methanol directly instead of hydrogen. Therefore, it has received great attention in applications for power supplying systems for mobile or portable devices [1,2,3,4,5,6,7].

However, despite the advantages described above, there are several problems such as low methanol oxidation kinetics concerning the use of a large amount of precious metals and methanol crossover from the anode to cathode side through the membrane [8,9,10,11]. Methanol crossover leads to the degradation of the performance such as a mixed potential by the reaction of crossed methanol at the cathode side, a CO poisoning of platinum particles and fuel loss. It should be overcome in order to commercialize the DMFC. It is necessary to develop a highly active catalyst, a low methanol-permeable membrane and a methanol-tolerant oxygen reduction reaction (ORR) catalyst. The novel design of the electrode structure, electrode materials and membrane-electrode-assembly (MEA) fabrication technique are also required to achieve it [12,13,14].

In this study, we first synthesized the nitrogen-doped carbon nanofiber (N-CNF) as the carbon support by the thermal decomposition of acetonitrile. Sixty wt % palladium-enriched platinum-palladium (Pt-Pd) catalysts on the carbon black (CB) or the N-CNF were prepared and compared to commercial Pt catalyst on the CB as a cathode material of the DMFC. The N-CNF has ORR activity itself by doping nitrogen. Further, nitrogen atoms in the carbon structure are also expected for their role in modifying electron negativity (C: 2.5, N: 3.0, precious metal Pt, Pd: 2.2) and inducing stronger bonding between deposited metals and carbon supports. Also, the d-band electronic vacancy due to the high electro-negativity of the N-CNF can be blocked by CO adsorption on the Pt particles. Therefore, Pt-Pd binary catalysts with methanol-tolerant ORR activity using the N-CNF were prepared and evaluated. Pt is already a well-known novel metal having the highest activity for ORR. Pd has a significant ORR activity, comparable with Pt, and a CO tolerance simultaneously. Pd is still an expensive metal but not compared to Pt. In addition, Pd is expected to have a good stability with Pt because of their similar valence shell electronic configuration and lattice constant [15,16,17,18].

## 2. Experimental Section

### 2.1. Preparation of Pt-Pd Catalyst

The N-CNF was prepared to react the vaporized acetonitrile over the nickel-iron catalysts supported on the magnesium oxide at high temperature as in previously reported experiment [19]. Acetonitrile was purged with nitrogen for 20 min to eliminate oxygen component before the experiment. The N-CNF was obtained by above reaction for 2 h at 640 °C and further treated in 10 wt % hydrochloric acid solution for 48 h to completely remove the metallic catalysts. The N-CNF and commercial carbon black (CB, Vulcan XC 72r, Cabot, Boston, MA, USA), which was chosen to compare the effect of carbon support material, were placed in 10 wt % of sulfuric acid solution for 2 h at 80 °C with reflux system, to introduce an oxygen functional group on the carbon surface.

The Pt-Pd binary catalysts were prepared by the impregnation method using NaBH_4_. The reagent ratio of Pt and Pd was set to be the weight ratio of 1:2. Specified amount of H_2_PtCl_6_·6H_2_O, PdCl_2_ and the carbon support materials were mixed in 400 mL of distilled water for 24 h. Then, NaBH_4_ aqueous solution of 400 mL, the amount of 15 times of metal molecule [20], was poured in Pt-Pd-carbon mixture with vigorous stirring. After 1 h, Pt-Pd catalysts supported on carbon were filtered and washed with distilled water several times. A commercial pure Pt catalyst supported on the CB (HiSPEC 9000, Johnson Matthey, London, UK) was chosen to compare the catalytic performance.

### 2.2. Characterization of Supporting Carbons

Morphologies and graphite stacking structure of the N-CNF were observed by scanning electron microscope (SEM, S-4700 Hitachi, Tokyo, Japan) and transmission electron microscope (TEM, Tecnai G2 F30, FEI, Hillsboro, OR, USA). Nitrogen adsorption isotherm curves at liquid nitrogen temperature were used to obtain the specific surface area of carbons by Brunauer, Emmett and Teller (BET) calculation (BELSORP-mini, BEL Japan Inc., Osaka, Japan). The content of doping nitrogen was examined by elemental analysis (FLASH EA-2000, Thermo Scientific, Waltham, MA, USA). Electrochemical measurements for capacitance and ORR activity were conducted using a conventional three-electrode system. A carbon ink was prepared following recipe. A mixture of 5 mg of carbon, 20 μL of distilled water, 800 μL of propyl alcohol and 85.8 μL of 5% Nafion solution was blended using a sonic bath and magnetic stirrer for 24 h. The working electrode was manufactured by loading of 5 μL of carbon ink and dryness. Cyclic voltammetry was performed with scan rate of 10 mV/s in Ar purged 0.1 M HClO_4_ electrolyte. After the electrolyte was purged oxygen for 20 min, the linear sweep was conducted to search the ORR activity with rotating disk electrode at 1600 rpm.

### 2.3. Characterization of the Pt-Pd Catalysts

The amount of loaded metal on Pt-Pd catalyst was analyzed via Thermogravimetry (TG) analysis (STA409PC, NETZSCH). Since Pt-Pd catalysts have very high reactivity for increased temperature, a heating rate was adjusted to 2 °C/min up to 800 °C. The composition ratio of Pt and Pd was identified using energy dispersive X-ray fluorescence (EDXRF) analysis (SEA 1200VX, Seiko, Chiba, Japan). The Scherrer formulate using X-ray diffraction (XRD) pattern (RINT 2000, Rigaku, Tokyo, Japan) of 65° to 71° peak region was adopted to estimate the particle size. Electro-catalytic activity for ORR was examined by conventional three electrode system same as the carbons. A catalyst paste was prepared that 10 mg of catalysts, 20 μL of distilled water, 1.6 mL of isopropyl alcohol and 57.2 μL of 5% Nafion solution were blended in sonic bath. The working electrode was manufactured by uploading the 5 μL of catalyst paste onto the mirror polished glassy carbon and completely dried. Oxygen purged 0.1 M HClO_4_ was used as electrolyte. The linear sweep in the range of 0.25 to 1.25 V vs. RHE was conducted with scan rate of 5 mV/s using rotating disk electrode at 1600 rpm. The same ORR experiment procedure to evaluate the methanol-tolerant was performed in oxygen purged 1 M MeOH + 0.1 M HClO_4_ electrolyte.

### 2.4. DMFC Unit-Cell Measurements

Membrane electrode assembly (MEA) was manufactured using a commercial Nafion 115 sheet (Nafion^®^, DuPont™, Wilmington, DE, USA) as a membrane. Conventional Pt-Ru catalyst (HiSPEC 12100, Johnson Matthey, London, UK) was chosen as an anode electrode material. PTFE treated carbon paper (TGP-H-010, Toray Carbon, Tokyo, Japan) was used as the gas diffusion layer (GDL) of the anode side. The cathode GDL was used to SGL carbon paper with microporous layers (SIGRACET GDL 25 BC, SGL Carbon, Wiesbaden, Germany). The active area of MEA was 9 cm^2^. The catalyst layer was prepared on the GDL by brushing with catalyst paste, which is composed of catalysts and Nafion ionomer well-dispersed in water and alcoholic based dispersant. The loading amount of the anode side was fixed at 3 mg/cm^2^ on the basis of metal weight. On the cathode side, metal based 1.2 mg/cm^2^ of catalysts was loaded. A hot-press with 25 kg/cm^2^ at 150 °C for 1 min was carried out to attach each electrode to membrane.

The performance of the MEAs with Pt-Pd catalysts as cathode electrode was obtained with an electro-chemical measurement system (WonATech Ltd., Seoul, Korea). I-V polarization curves for MEA performance were measured by the current step mode in a state where the supplement of air of 400 mL/min to the cathode side. The amount of methanol solution on anode was adjusted to the fixed amount of pure methanol based on the 3 mL/min of 1 M methanol solution. Methanol solution concentration was increased to 5 M and reduce the flow rate to feed a constant amount of pure methanol. Meanwhile, in order to evaluate the stable operation with high concentration, constant current of 50 mA/cm^2^ to maintain usual DMFC operating voltage of over 0.4 V was conducted for 2 h. Air was supplied to the cathode electrode at a flow rate of 80 mL/min (10 times of the theoretical demand), and 5 M methanol solution was fed to anode at a flow rate of 0.047 mL/min (five times of the theoretical demand). The measurements were performed at 60 °C. An average potential of an hour later was collected to compare the stable catalytic performance.

## 3. Results and Discussion

### 3.1. Characterization of the N-CNF

**Figure 1:**
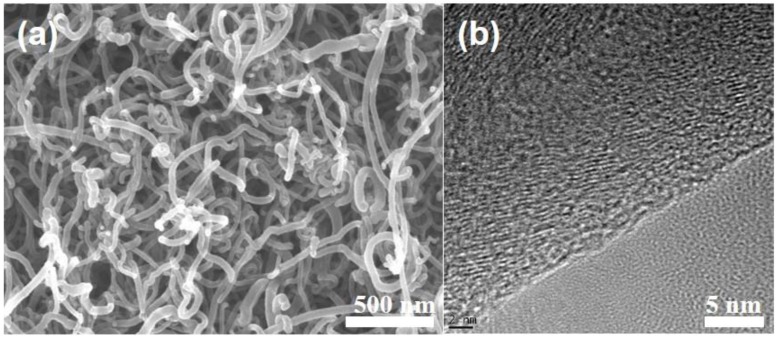
The scanning electron microscope (SEM) and transmission electron microscope (TEM) images of the nitrogen doped carbon nanofiber (N-CNF).

Figure 1 is the SEM and TEM images that show the morphology and graphitic stacking structure of the N-CNF, respectively. A fiber diameter in the range of 20 to 30 nm was observed in the N-CNF synthesized at 640 °C. The TEM image indicates that a well-aligned fish-bone (herringbone)-type graphitic stacking structure, with the edge of the graphene layer exposed on the surface, was discovered in the N-CNF. The core part of the N-CNF also appeared partially empty as in a bamboo-type shape.

Characteristic differences of the N-CNF and CB were summarized in Table 1. The 8.4 atomic percent of nitrogen (the ratio of nitrogen moles divided by the total number of moles of nitrogen and carbon) was confirmed via elemental analysis in the N-CNF. The specific surface area of the N-CNF showed a slightly lower value than the CB. However, the specific capacitance of the N-CNF calculated from the cyclic voltammogram appeared at a higher value of 38.1 F/g in comparison with the CB value of 33.6 F/g. This may be because of the stronger interaction between the ions in the electrolyte and the modified surface of the N-CNF by the enhanced electron negativity [21].

**Figure 2:**
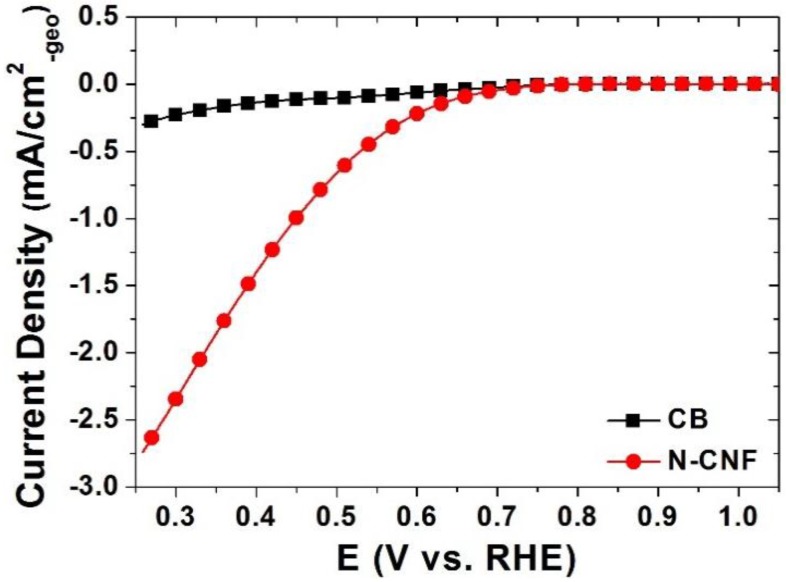
Presence of oxygen reduction reaction (ORR) activity in the N-CNF measured by conventional electrochemical equipment.

Figure 2 shows the ORR activity of the carbons with a rotating disk electrode. Although the limiting current density of the N-CNF was not detected, the ORR activity displayed nearly 0.75 V despite no detection of the CB. It was confirmed that the N-CNF had a unique characteristic for electrochemical behavior, and it could have a considerable effect on the activity and durability when used as a catalyst support [22,23,24].

### 3.2. Characterization of the Pt-Pd Catalysts

**Figure 3:**
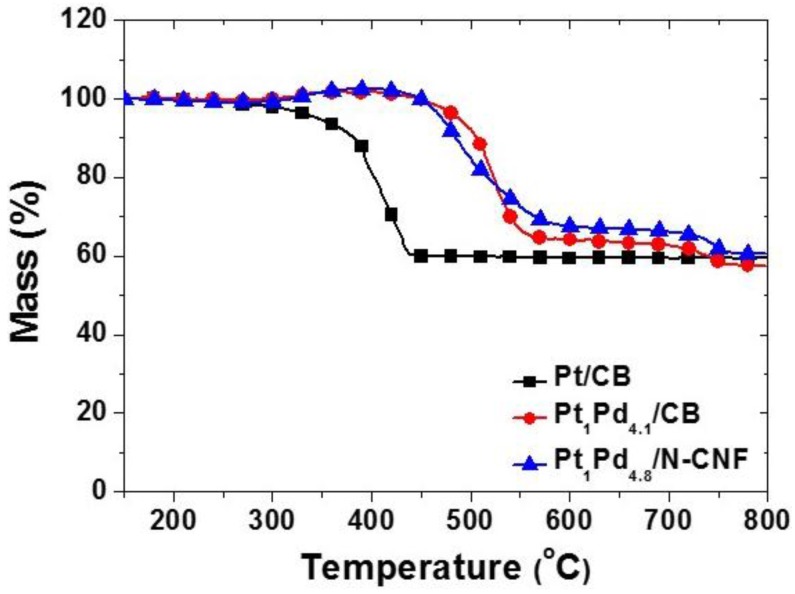
Thermogravimetry (TG) profiles of commercial Pt catalyst and Pt-Pd binary catalysts on the carbon black (CB) or the N-CNF.

Figure 3 explains the thermogravimetry reflections of each 60 wt % metal catalyst. The Pt-Pd binary catalysts had different profiles from the commercial pure Pt catalyst. The slight weight increase was found at 300 °C due to the oxidation of the catalyst, and the weight loss started at 450 °C for the Pt-Pd binary catalysts. The stabilized weight change around 600 °C was dropped again near 750 °C. Even if the thermal decomposition of the N-CNF was begun at a lower temperature than the CB due to the action of nitrogen as a pro-oxidant, the gradual decline slope appeared in the N-CNF. It was because the nitrogen content of the N-CNF promoted the onset of oxidation, and the fibrous and well-aligned graphitic structure of the N-CNF displayed outstanding oxidative resistance compared to the CB with a nano-particle cluster.

The amount of loaded metal from the TG residual data and the weight ratio of Pt-Pd from the EDXRF analysis are summarized in Table 2. Although the reagent ratio of Pt and Pd was set as the weight ratio of 1:2 in the catalyst synthesis, a palladium-rich surface was detected by the EDXRF. From the TG analysis, the targeted 60 wt % Pt-Pd binary catalysts were successfully prepared using the NaBH_4_ impregnation method. As a result of these experiments, the Pt-Pd binary catalysts are predicted to have a core-shell structure of Pt-Pd nanoparticles. The Pt-Pd catalysts with a palladium-rich surface composition were referred to as Pt_1_Pd_4.1_/CB and Pt_1_Pd_4.8_/N-CNF, respectively. The particle size of Pt-Pd is listed in Table 2 and was derived using the Scherrer equation from a peak value of about 68° of the (220) peak. The particle size of the Pt-Pd catalysts was smaller than the commercial pure Pt catalyst. Although the Pt-Pd catalysts were synthesized under the same conditions, the metal composition and particle size were different according to the carbon support material. Using the N-CNF as a support, there was small particle size and the surface composition was palladium-rich. It was assumed that there was an electronic effect caused by the modification of introduced nitrogen in the carbon structure [25], but additional studies should be conducted to obtain further understanding.

**Figure 4:**
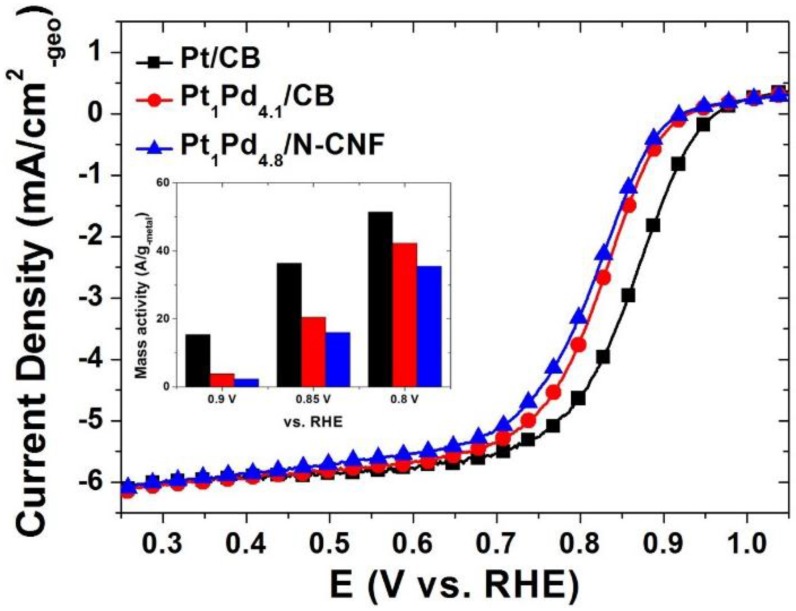
Activity for ORR of the catalysts in 0.1 M HClO_4_ with a rotating disk electrode at 1600 rpm.

**Figure 5:**
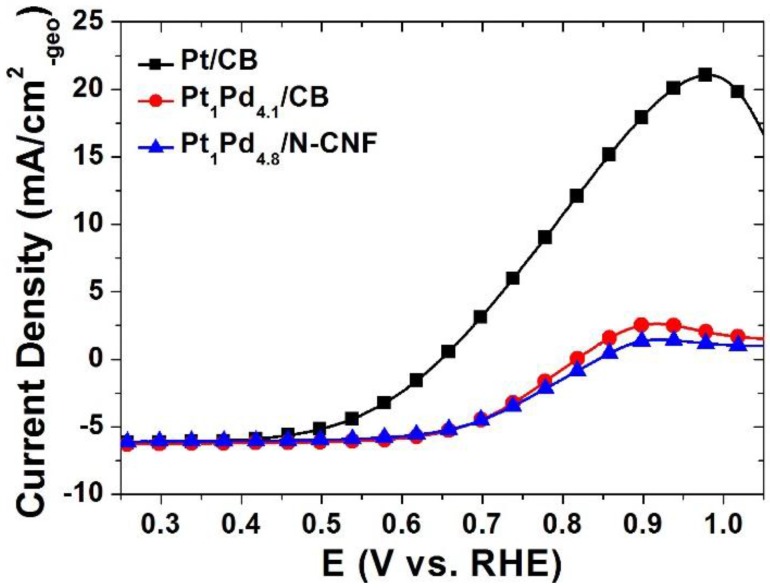
Activity for ORR of the catalysts in presence of 1 M methanol with oxygen.

Figure 4 shows the ORR activity of the catalysts. The ORR activity of the commercial Pt catalyst occurred at 0.97 V. In case of the Pt-Pd catalysts, however, the onset potentials for ORR were shifted in the negative direction with the increased palladium constituent. The potential at the half-wave current is stated in Table 3. The smaller amount of Pt in the whole catalyst brought about the low value of the half-wave potential in the same terms of high over-potential. In accordance with our experiment, the most critical factor of ORR activity has been typically the amount of platinum. In normal conditions, it was not enough to compensate for the activity of platinum by palladium. Figure 5 indicates the ORR activity in the presence of methanol in the electrolyte. The typical shape of the current behavior for methanol oxidation was recorded in the commercial Pt/CB catalyst. On the other hand, the Pt-Pd binary catalysts had the current curves with a general ORR activity without the participation of methanol. Pt_1_Pd_4.1_/CB appeared to suffer a bit due to methanol oxidation in the region of more than 0.8 V, but the methanol oxidation peak was much less likely to occur in the Pt_1_Pd_4.8_/N-CNF. As a result of the above experiment in the presence of methanol, a methanol-tolerant ORR activity of the Pt-Pd catalysts was obviously confirmed, and the N-CNF as the carbon support material gave an impulsion to it.

### 3.3. Unit-Cell Performance for DMFC

**Figure 6:**
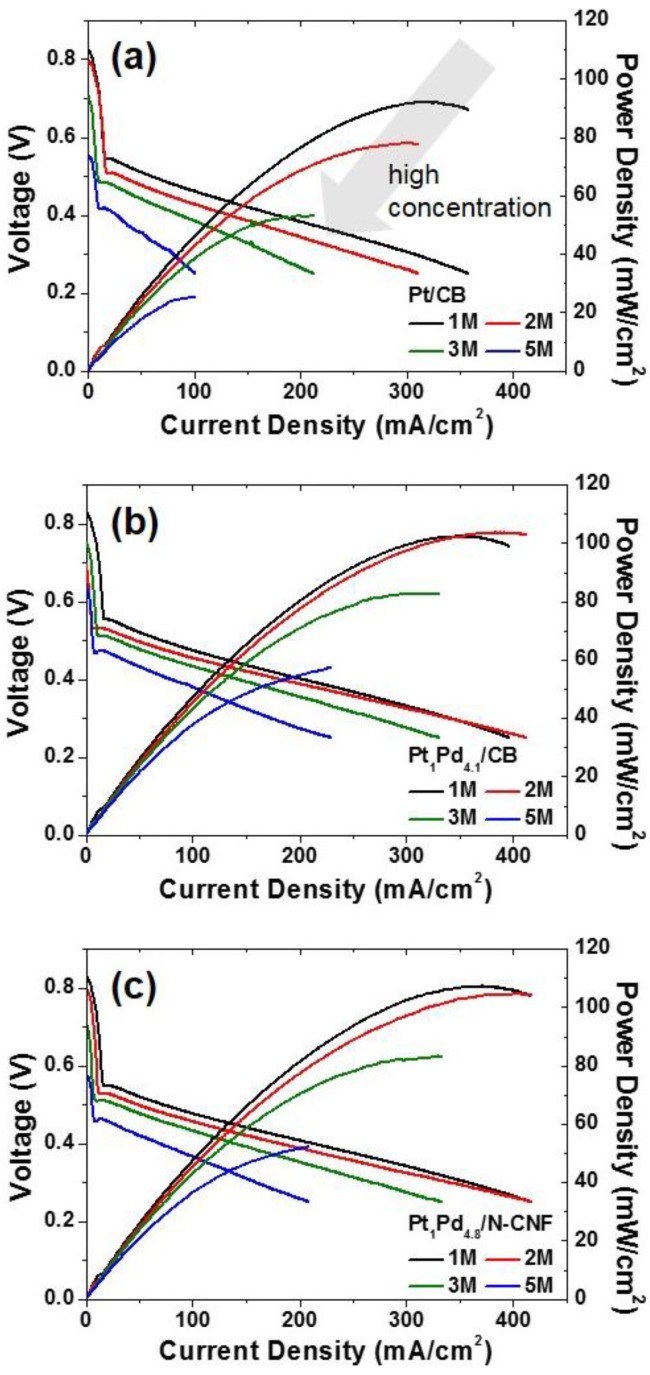
The direct methanol fuel cell (DMFC) unit-cell performances of the membrane electrode assemblies (MEAs) prepared with the catalysts of (**a**) commercial Pt/CB; (**b**) Pt_1_Pd_4.1_/CB and (**c**) Pt_1_Pd_4.8_/N-CNF with various methanol concentrations.

The I-V polarization curves and power densities of the DMFC unit-cell measurements with the above cathode electrode materials at 60 °C using various methanol concentrations are expressed in Figure 6. In the commercial catalyst, there was a lower performance as the concentration of the feed methanol solution increased due to the mixed potential and CO poisoning of the catalyst by methanol crossover. Even if the performance reduction was found in both Pt_1_Pd_4.1_/CB and Pt_1_Pd_4.8_/N-CNF, the methanol-tolerant cathode catalysts maintained a significant performance compared to the commercial catalyst even with a 1 M methanol feed.

**Figure 7:**
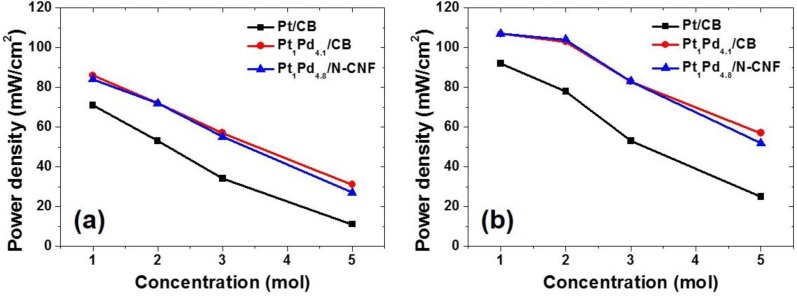
Comparisons of power densities at (**a**) 0.4 V and (**b**) maximum peak point.

Figure 7 shows the power densities of the DMFC unit-cell experiments at 60 °C according to the concentration of feed methanol. Figure 7a indicates the performance at 0.4 V, and Figure 7b compares with the maximum peak performance. The Pt-Pd binary catalysts had a higher power density than that of the commercial Pt catalyst in all experimental conditions. Even in the general operating condition of using 1 M methanol, the Pt-Pd catalysts had enhanced values compared to the pure Pt catalyst. Although the ORR activity was increased with the amount of Pt in the electrochemical tests, the pure Pt catalyst was presented to be diminished in the actual unit-cell test. It was because the effect of methanol crossover occurred at 1 M methanol as a relatively low concentration. Therefore, the high performance of the unit-cell test appeared with the Pt-Pd catalysts in all conditions with the methanol concentration. There were similar power densities except with the high concentration of 5 M methanol. When the 5 M methanol solution was fed, Pt_1_Pd_4.1_/CB had the best power density both at 0.4 V and the peak point.

**Figure 8:**
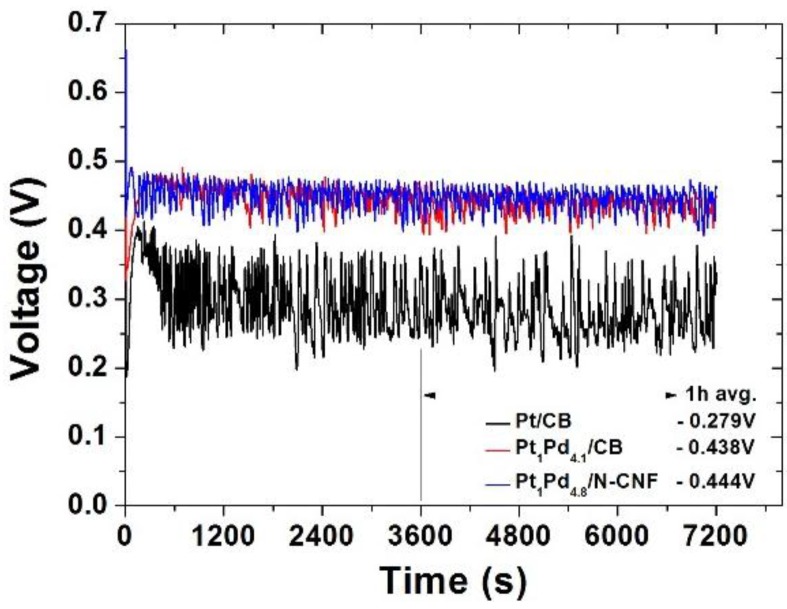
Constant current operation of the MEAs with the catalysts as cathode materials.

The variation of the voltage with the applied constant current of 50 mA/cm^2^ was recorded as shown in Figure 8. Likewise, in the I-V polarization curves, the Pt-Pd binary catalysts retained the higher voltage. The commercial catalyst appeared to have a very large voltage variation in the range of 0.2 and 0.4 V. It seems to be due to the susceptible reaction in the repetition of CO poisoning and the re-activation of the platinum particles caused by the undesired methanol crossover. It can be also assumed that the Pt-Pd catalysts had highly catalytic activity and tolerance to CO poisoning simultaneously. The average voltages for the latter 1 h were 0.279 V of the commercial catalyst, 0.438 V of Pt_1_Pd_4.1_/CB and 0.444 V of Pt_1_Pd_4.8_/N-CNF, respectively. The temporary performance of the I-V polarization curves by current step scanning was slightly higher in Pt_1_Pd_4.1_/CB, but Pt_1_Pd_4.8_/N-CNF was shown to have a more stable performance in the constant current mode test. In view of the fact that the constant current operation is applied for the actual DMFC system, Pt_1_Pd_4.8_/N-CNF is thought to be a more appropriate selection as the cathode electrode catalyst of the DMFC system using a high concentration of methanol. Furthermore, although the constant current test was conducted for the short duration of 2 h, the durability of the above catalysts could be discussed on the basis of the experimental results and previous reports [25,26]. The electron negativity of the N-CNF as the carbon support could be modified at a higher value (C: 2.5, N: 3.0, Pt and Pd: 2.2) by introduced nitrogen. It is confidently expected that Pt-Pd on the N-CNF possesses good durability and activity due to a stronger bonding between the carbon support and the catalytic metal particles by the increased electron negativity of the carbon support and the enhanced electric conductivity of the N-carbon. From the above results, the Pt_1_Pd_4.8_/N-CNF catalyst is a promising candidate as a cathode material for the DMFC system using a high concentration of methanol.

## 4. Conclusions

Pt-Pd catalysts supported on the novel nitrogen-doped carbon nanofibers (N-CNF) were prepared and evaluated for the methanol-tolerant oxygen reduction reaction (ORR). The N-CNF, which was directly synthesized by catalytic chemical vapor deposition from acetonitrile, was verified to have a change of electrochemical surface properties such as oxygen reduction reaction activities and the electrochemical double layer compared to common carbon black (CB). To attain the competitive oxygen reduction reaction activity and methanol tolerance, the (Pd-enriched) Pt-Pd metals were loaded on the N-CNF and the CB. The physical properties of the N-CNF or the CB supported Pt-Pd catalysts were examined. To evaluate the electrochemical characteristics, activities for ORR and ORR in the presence of methanol tests were conducted and compared with a commercial Pt catalyst. Also, the unit-cell of direct methanol fuel cell (DMFC) using these catalysts as cathode electrode materials was applied to obtain the I-V polarization performance with the methanol concentration. The constant current test also evaluated the stable performance in a high concentration of methanol. On the basis of the experiment results, the Pt-Pd binary catalysts had a methanol-tolerant ORR activity and could retain the performance in a high concentration in the DMFC test. When the N-CNF was used as the catalyst support material, a good performance over a long duration with a high-concentration feed methanol is confidently expected. These methanol-tolerant ORR catalysts may facilitate the use of a higher-concentration methanol, and may also enable higher energy density and a simplified system.

## Figures and Tables

**Table 1:** Characteristics of carbon support materials.

Sample	Nitrogen (%)	Specific Surface Area (m^2^/g)	Capacitance (F/g)
CB	-	222	33.6
N-CNF	8.4	147	38.1

**Table 2:** Composition and crystallite size of the catalysts.

Sample	Metal (wt %)	Pt:Pd Weight Ratio	Crystallite Size (nm)
**Pt/CB**	59.0	Only Pt	4.5
**Pt-Pd/CB**	57.5	1:4.1	3.7
**Pt-Pd/N-CNF**	60.6	1:4.8	3.4

**Table 3:** Comparison of half-wave potential from ORR activity measurement.

Sample	Half-Wave Potential (V)
Pt/CB	0.862
Pt_1_Pd_4.1_/CB	0.825
Pt_1_Pd_4.8_/NCNF	0.814
